# Nano Porous Carbon Derived from Citrus Pomace for the Separation and Purification of PMFs in Citrus Processing Wastes

**DOI:** 10.3390/nano10101914

**Published:** 2020-09-25

**Authors:** Zhenqing Li, Xin Chen, Lulu Qiu, Yu Wang, Zhiqin Zhou

**Affiliations:** 1 College of Horticulture and Landscape Architecture, Southwest University, Chongqing 400716, China; zhenqinglee@126.com (Z.L.); chenxinruyi676893@163.com (X.C.); 18696516978@139.com (L.Q.); wyoo20@163.com (Y.W.); 2 The Southwest Institute of Fruits Nutrition, Banan District, Chongqing 400054, China; 3 Key Laboratory of Horticulture Science for Southern Mountainous Regions, Ministry of Education, Chongqing 400715, China

**Keywords:** citrus pomace, citrus nanoporous carbon, PMFs, adsorption/desorption

## Abstract

The by-product of citrus juice processing is a huge source of bioactive compounds, especially polymethoxyflavones (PMFs) and fibers. In this study, a method for the separation and purification of PMFs from citrus pomace was established based on citrus nanoporous carbon (CNPC) enrichment. Different biomass porous carbons were synthesized, their adsorption/desorption characteristics were evaluated, and the CNPCs from the peel of *Citrus tangerina* Tanaka were found to be best for the enrichment of PMFs from the crude extracts of citrus pomace. Using this method, six PMF compounds including low-abundant PMFs in citrus fruits such as 5,6,7,4′-tetramethoxyflavone and 5-hydroxy-6,7,8,3′,4′-pentamethoxyflavone can be simultaneously obtained, and the purities of these compounds were all higher than 95%, with the highest purity of nobiletin reaching 99.96%. Therefore, CNPCs have a great potential for the separation and purification of PMFs in citrus processing wastes, potentially improving the added value of citrus wastes. We also provide a method reference for disposing of citrus pomace in the future.

## 1. Introduction

Citrus fruits rich in various secondary metabolites are the most consumed fruits in the world because of their nutritional value and health benefits [1]. The citrus processing products are also popular among consumers. Approximately one third of citrus fruits are utilized for processing, which produces 50–60% organic waste, causing great waste and environmental pollution [1,2]. Traditional methods of citrus processing waste (CPW) disposal (such as land filling or incineration) are insufficient and problematic in terms of environmental impacts and energy efficiency [3,4]. Applications using the whole citrus peel without differentiating individual constituents as animal feed, organic fertilizer, and base for compost represent the most common and simplest way to process waste citrus materials [5]. Since CPW contains many high-value-added compounds such as soluble sugars, cellulose, pectin, essential oils (EOs), and polymethoxyflavones (PMFs), biorefining can valorize this agricultural processing waste while producing fewer to-be-treated wastes [5]. Ángel et al. and Boukroufa et al. [6] have proposed extracting EOs, polyphenols, and pectin from CPW with microwaves and ultrasound-assisted extraction. D-limonene and α-terpineol were obtained in the scheme proposed by Pourbafrani et al. [7] and Balu et al. [8]. Satari et al. [1] and Zhang et al. [9] reported means of CPW valorization: the direct use of native/modified CPW for producing nanomaterials, such as carbon-based Fe_3_O_4_ nanocomposites (C/Fe_3_O_4_ NCs). Additionally, there are studies that have reported that flavonoids such as hesperidin and naringin were obtained from citrus pomace by subcritical water extraction [10], but few articles have mentioned the availability of PMFs in citrus processing wastes.

Polymethoxyflavones are a group of flavonoids that exist widely in citrus fruits, particularly in the peels of *Citrus reticulata* Blanco and *Citrus sinensis* Osbeck—always wasted as CPW—which possess the highest amounts of PMFs compared to other edible parts of the fruit [11,12]. Compared with other flavonoids in CPW, PMFs present a better bioavailability and have been studied for improving skeletal muscle mitochondrial biogenesis, for their anti-diabetes and weight control effects, and for their antioxidant activity [13,14]. More importantly, PMFs have gradually been used to prevent and treat various human chronic diseases [15], or as bioavailability enhancers to enhance the biological activity of drugs [16]. However, the in vivo study of PMFs has been performed only in a handful of rodents, because the supply of a large quantity of pure PMFs is the bottleneck step. Although some PMFs are commercially available, the cost is too high to perform human efficacy studies [17,18]. Currently, silica gel column chromatography was the traditional preparation method for PMFs from citrus peel [19]. It is widely used but complicated to operate, and steps are tedious. High-speed counter-current chromatography (HSCCC) or high-performance counter-current chromatography (HPCCC) have also been used to prepare PMFs, but are insufficient for the desired availability of sufficient phytochemicals in current fast-pace research both in pharmaceuticals and nutraceuticals [18]. Macroporous adsorption resins (MARs) have been widely used due to their high adsorption/desorption capacity and low cost, but the safety of MARs in the food and pharmaceutical industry remains a major concern [20].

The increasing demand for contamination-free food products with the growth of the population and the development of technology are raising serious ecological and environmental issues associated with food safety. Activated carbon (AC) and some 3D carbon adsorbents, such as graphene aerogel (GA), usually derived from bio-substances (e.g., citrus pomace, pectin, or coconut shell), are forms of carbon processed to have a high surface area and proper pores, and further chemical treatment often enhances the adsorption properties. In the past few decades, the application of biomass-based GA and ACs for environmental remediation and food security has received much attention due to the easy processing of their natural sources, low cost, and reduced environmental footprint [21]. GAs as adsorbents have shown their versatility as pollutant absorbers for the removal of dyes, formaldehyde, and other environmental toxins; on membranes for separating oil-in-water emulsion; and for efficient food toxin removal [22,23]. AC is often used as an adsorbent to remove dark-colored compounds [24], heavy metals [25], pesticides [26], and some organic acids [27,28], but few reports exist on the use of AC to enrich PMFs from CPW. In the adsorption process of AC, it is simpler and less time-consuming when compared to other methods. ACs have a high mechanical strength, have a good acid and alkali resistance, and are also stable and easy to maintain. When compared with other ACs, citrus nanoporous carbons (CNPCs) present a better adsorption/desorption efficiency, which is more suitable for the enrichment of PMFs. Considering the advantages and disadvantages of various separation methods, CNPCs may provide a promising choice for preparing PMFs. 

Hence, in this study CPWs were employed to extract PMFs and prepare CNPCs. Subsequently, CNPCs were used as adsorbents to enrich PMFs and combined with an existing MS-directed prep-HPLC method [19] for the separation and purification of PMF compounds. Lastly, the method for the separation and purification of PMFs from citrus pomace was proposed based on citrus nanoporous carbon enrichment, which has great potential for isolating and purifying PMFs in citrus pomace.

## 2. Materials and Methods

### 2.1. Chemicals and Materials

The PMF extraction was prepared from citrus pomace (*Citrus sinensis* Osbeck) provided by Chongqing Fresh Fruit Orange Juice Co., Ltd (Chongqing, China). Analytical-grade ethanol was purchased from Chengdu Kelong Chemical Reagent Co., Ltd. (Chengdu, China); hydrochloric acid and other chemicals were all purchased from Xiangyue Chemical Co., Ltd. (Chongqing, China); formic acid and methanol (MS grade) were purchased from Sigma-Aldrich (St, Mo, USA); and distilled water was made by Milli-Q Advantage A10 (Millipore Co., Ltd., MA, USA). The detailed information of the main flavonoid standards used in this study is listed in Table S1.

The precursor sources for nanoporous carbon synthesis were different agricultural processing wastes, including citrus pomace after juice processing, zanthoxylum waste, and olive residue after squeezing oil. All of these wastes come from various processing factories. The commercial activated carbon was purchased from Rhawn Chemical Reagent Co., Ltd. (Shanghai, China).

### 2.2. Synthesis and Characterization of the Carbon Adsorbents

The processing waste-derived nanoporous carbons (freeze-dried citrus nanoporous carbon: CNPC1; zanthoxylum porous carbon: ZPC; olive porous carbon: OPC; air-dried citrus nanoporous carbon: CNPC2; citrus nanoporous carbon-based Fe_3_O_4_ composites: MCNPC) were synthesized in a typical process, as reported in previous literature, with some modifications [29]. Briefly, processing wastes, porogen, and ultrapure water (1:4:100, *w*/*w*/*v*) were mixed and stirred for about 8 h at 40 °C. Subsequently, the mixture was air-dried at 40 °C or freeze-dried to a constant weight and then transferred to a tube furnace and heated at 500 °C under nitrogen protection for 2 h. Then, the as-prepared samples were washed with 1M of HCl and deionized water, respectively, to remove any inorganic impurities such as porogen ZnCl_2_ until the pH value was about 5. Finally, the products were dried at 50 °C.

A Hitachi S–4800 field-emission scanning electron microscope (SEM, Tokyo, Japan) operated at 20 kV was used to obtain the morphology of the prepared carbons. X-ray diffraction (XRD) analyses were performed by a Bruker D8 diffractometer (Bruker, Karlsruhe, Germany) using the CuKa radiation. Fourier transform-infrared (FT–IR) spectra were recorded using a Nicolet iS10 FT–IR spectrophotometer (Thermo Fisher, Waltham, MA, USA).

### 2.3. Preparation of Crude Extracts from Citrus Processing Wastes

In the present study, PMFs were extracted in a factory using a heat reflux extraction method according to our previous reports with modifications [19]. The CPWs were dried in air at room temperature and crushed to a particle size of 3–4 mm, then extracted using an 90% (*v*/*v*) ethanol-water solution at 40 °C for 3 h; this was repeated twice. The supernatant was collected and combined, and then concentrated in vacuum at 45 °C to get a crude extract of total PMFs. The concentration of the sample solution was determined by ultra-high performance liquid chromatography (UPLC) analysis.

### 2.4. UPLC Analysis of PMFs

The PMFs content was determined using ultrahigh-performance liquid chromatography (UPLC, Waters ACQUITYI-Class (Milford, MA, USA) equipped with a photodiode array detector, a quaternary solvent delivery system, and a column temperature controller). Chromatographic separations were performed on a 2.1 × 100 mm, 1.7 µm ACQUITY UPLC HSS C18 column (Waters, MA, USA). The mobile phase consisted of water/formic acid (99.99%: 0.01%, *v*/*v*) (A) and methanol (B) at the rate of 0.4 mL/min, and the gradient profile was as a previous study reported [19]. All the prepared samples were tested in triplicate.

### 2.5. Optimization of the Enrichment Process

#### 2.5.1. Adsorption and Desorption Properties of Activated Carbons

The adsorption/desorption properties of porous carbons were detected as follows: carbons (around 0.1 g) and 50 mL of PMF solution with a known concentration (C_0_) were added into 150 mL flasks with stoppers for 12 h, then filtered, and the carbons were washed with pure water to remove superficial PMFs. Next, the carbons were desorbed 3 times with 50 mL of 95% ethanol, the flasks were shaken with a speed of 130 rpm at 25 °C for 12 h, and the filtrate concentrations (C_1_) and final desorption concentrations (C_2_) were analyzed by UPLC. The corresponding adsorption and desorption ratios of each carbon were calculated using the equations below:

(1) $$\mathrm{Adsorption}\;\mathrm{capacity}\;\left(\mathrm{mg}/g\right)\;=\;\frac{\left(C_{0}-C_{1}\right)\times 50}{0.1},$$

(2) $$\mathrm{Desorption}\;\mathrm{capacity}\;\left(\mathrm{mg}/g\right)\;=\;\frac{C_{2}\times 50}{0.1},$$

(3) $$\mathrm{Desorption}\;\mathrm{ratio}\;=\;\frac{C_{2}\times 50}{\left(C_{0}-C_{1}\right)\times 50}\times 100\%.$$

#### 2.5.2. Adsorption and Desorption Kinetics of the Selected Porous Carbon

The kinetics curve for PMFs on the selected carbon was studied by mixing 160 mL of the sample solutions with 0.5 g of carbon in 500 mL flasks. The flasks were then shaken (130 rpm) at 25 °C for 24 h. The concentrations of PMFs in the adsorption/desorption solution were determined at different time intervals. Adsorption kinetics are usually analyzed using three models, including pseudo-first-order (4), pseudo-second-order (5), and intra-particle diffusion kinetics models (6). The models can be expressed by the following mathematical formulas:

(4) $$Q_{t}=Q_{e}-Q_{e}e^{k_{1}t},$$

(5) $$Q_{t}=\frac{k_{2}Q_{e}^{2}t}{1+k_{2}Q_{e}t},$$

(6) $$Q_{t}=k_{3}t^{\frac{1}{2}}+c,$$

where Q_t_ (mg/g) is the concentration of solute per mass of adsorbent at time t; k_1_, k_2_, and k_3_ are the pseudo-first-order, pseudo-second-order, and intra-particle diffusion rate constants, respectively; c is a constant.

#### 2.5.3. Adsorption Isotherms on the Selected Activated Carbons

The carbon adsorbents (0.1 g) and 50 mL of the aqueous solution of the CPW extract were added into a 150 mL flask. The initial PMFs concentrations ranged from 100 to 1200 mg/L. The flasks were shaken at 25 °C until an equilibrium adsorption was achieved. The PMF concentrations were tested at different intervals until equilibrium. The isotherms were studied at different temperatures (25–45 °C). The model of Langmuir (7) and Freundlich (8) can be expressed as follows:

(7) $$Q_{e}=\frac{Q_{m}K_{L}C_{e}}{1+K_{L}C_{e}},$$

(8) $$Q_{e}=K_{F}C_{e}^{\frac{1}{n}},$$

where Q_e_ is the same as described above; C_e_ (mg/mL) is the equilibrium concentration of PMFs in the solutions; K_L_ and K_F_ are the associated Langmuir and Freundlich constants, respectively; Q_m_ (mg/g) is the maximum adsorptive capacity, theoretically.

#### 2.5.4. Experimental Design for Optimization of PMFs Enrichment Process

To screen optimal variables for preparing PMFs with a higher purity, variables including the pH value, concentration of total PMFs, adsorption time, desorption ethanol concentration, eluting volume, and eluting time were tested and analyzed by a static single-factor experiment. The six parameters above that were predicted to affect the adsorption/desorption rate of PMFs were investigated to select those with optimal influence. 

Pre-weighted CNPCs (0.1 g) were loaded into different flasks, then 50 mL of crude PMFs of pH 7 (initial concentration of 0.7 mg/mL) were added, the mixture was shaken for 6 h and filtered, and the content of remaining PMFs was measured. The crude extract of PMFs was filtered for 6 h after shaking, and the content of PMFs in the filtrate was determined. The liquid remaining in the CNPC was washed with distilled water. Then, 50 mL of 90% ethanol solution was added to the flasks. After desorption for 6 h, it was filtered and the content of eluted PMFs was measured. The variables were adjusted as follows: pH (pH = 3, 4, 5, 6, 7, 8, 9, 10, 11); loading concentration (0.47, 0.76, 1.07, 1.25, 1.53 mg/mL); adsorption time (0.5, 1, 1.5, 2, 2.5, 3, 4, 5, 6 h); desorption of ethanol concentration (10%, 20%, 40%, 60%, 70%, 80%, 90%, and 100%); desorption volume (10, 15, 20, 25, 30, 35, 40, 45, 50 mL).

### 2.6. Separation of Polymethoxyflavones by Prep-HPLC

The separation was based on our previous study and performed on a XBridge C18 column (19 × 250 mm, 5 μm) with the conditions as follows: acetonitrile-water (A) with 0.1% formic acid (B) was used as the mobile phase with a gradient elution (0–6 min, 18–30% A; 6–35 min, 30–42% A; 35–45 min, 42–60% A; 45–50 min, 60–18% A), the flow rate was 20 mL/min, and the injection volume was 650 μL. Peak fractions were collected automatically according to the retention time and the online-QDa detection. Each peak from the chromatogram was collected, evaporated under reduced pressure, and then analyzed by UPLC-Q-TOF-MS/MS and NMR as reported [19].

### 2.7. Crystallization

Different antisolvents (such as deionized water, n-hexane, and ethyl acetate) have different effects on PMFs crystallization, and a low temperature is more suitable for PMF crystallization. In this study, deionized water was used as the crystallization antisolvent for PMFs [30]. The eluent from each cycle of chromatography was dried in a rotary evaporator at −0.1 MPa and 50 °C, and dissolved in ethanol, then anti-solvent was added and crystallized at 4 °C. Crystals were filtered after 24 h and washed at the same temperature 2–3 times. Crystals were air-dried and analyzed by UPLC.

## 3. Results and Discussion

### 3.1. Characterization of Citrus Nanoporous Carbon

**Figure 1:**
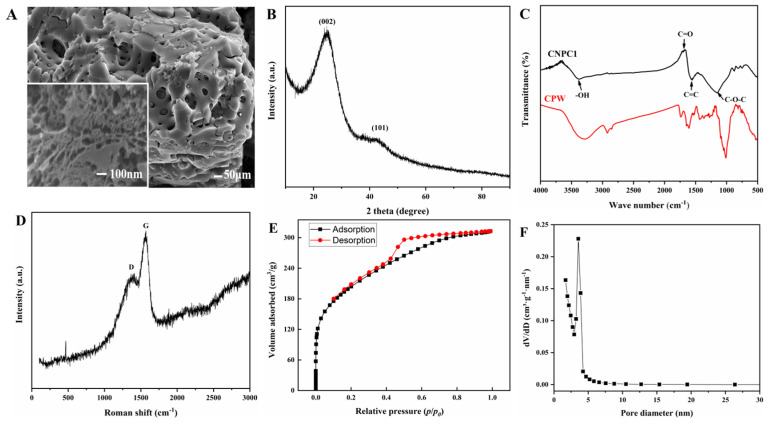
(**A**) SEM image, (**B**) XRD pattern, (**C**) FT–IR spectra, (**D**) Raman spectrum, (**E**) nitrogen adsorption isotherms, and (**F**) pore size distributions of citrus nano porous carbon.

The morphological features of the as-synthesized CNPC1 were investigated using scanning electron microscopy (SEM). The low-resolution SEM image of CNPC1 in Figure 1A shows that it has a highly porous and amorphous structure. The inset in Figure 1A, a high-resolution SEM image of CNPC1, also shows that it has a rich pore structure that endows it with a high surface area and porosity, and with numerous channels favorable for adsorption. Citrus wastes contain a large number of hydroxyl groups, and the activator ZnCl_2_ catalyzes the cracking of hydroxyl groups and promotes the decomposition of biopolymers at high temperatures [31]. Therefore, during the carbonization process the citrus wastes were dehydrated, resulting in a porous structure with the carbonization and aromatization of the carbon skeleton [32]. Additionally, the nitrogen adsorption-desorption isotherm in Figure 1E has a hysteresis loop, indicating that both microporous (0–2 nm) and mesoporous (2–50 nm) structures existed in CNPC1, with a pore size distribution in the range of 0–5 nm (Figure 1F). The specific surface area of CNPC1 is 723.5 m^2^/g, and the pore volume is 0.48 cm^3^/g. It is to be emphasized that these formed porous structures were favorable for entrapping PMF molecules and the rapid diffusion of PMF molecules in the surface of CNPC1. In addition, the morphology of CNPC1 after the adsorption/desorption process (Figure S1) was observed in a SEM image; it showed that the CNPC1 still maintained the porous structure. Therefore, we think that this material can effectively enrich PMFs, and also has a high structural stability, as previously reported in the literature [33].

In order to analyze the structure and phase of CNPC1, an XRD analysis was performed, as shown in Figure 1B. The two broad peaks at about 25.7° and 41.1° correspond to (002) and (101) reflections of the disordered carbon layer [34]. Additionally, the high intensity in the low-angle region might be due to the rich micropores in CNPC1 [29]. Figure 1C represents the FT–IR spectra of CNPC1 and CPW. As observed, the surface of CPW contains abundant functional groups, while they are reduced on the surface of CNPC1, but there is still a small amount of -COOH and -OH. In the FT–IR spectra of CNPC1, the peaks at 3424, 1702, 1645, and 1045 cm^−1^ correspond to -OH, C=O, C=C, and C–O–C, respectively. Additionally, the degree of crystallization of CNPC1 was studied by the Raman spectra. As shown in Figure 1D, the peaks at 1318 cm^−1^ (D band) and 1570 cm^−1^ (G band) were related to the lattice-defect carbon and graphitic carbon in CNPC1 [35], which is consistent with the XRD results. 

The above characterization results show that this material is a CPW-derived nanoporous carbon. Its micropores and mesopores can adsorb macromolecular substances. The high specific surface area and pore volume enable the adsorbent to fully contact the adsorbed molecules, and the functional groups on the surface further increase the contact. Therefore, CNPC1 could have a strong adsorption effect on active substances such as PMFs during the enrichment process. Similarly, an enrichment method for CGA using mesoporous carbon has been developed by Qin et al [28]. The mesopore volume of the carbon adsorbents was found to significantly influence the adsorption capacity. Among the carbon adsorbents tested, the MC3 is mainly composed of mesopores, so MC3 with a large mesoporous volume offers the highest adsorption capacity (294 mg/g of carbon), which is higher than those of other adsorbents reported in the literature. This may also apply to the process of PMFs adsorbed on CNPC1.

### 3.2. Static Adsorption and Desorption Capacities

The adsorption/desorption properties of six types of activated carbon were studied for the enrichment of PMFs. It could be clearly seen that the adsorption capacity (494.64 mg/g), desorption capacity (435.62 mg/g), and desorption ratio (88.07%) of PMFs on CNPC1 was considerably higher than on CNPC2 (490.84 mg/g, 419.42 mg/g, 85.45%) and OPC, ZPC, MCNPC, and AC (in Table 1). Thus, CNPC1 was selected as the most suitable adsorbent to enrich PMFs and used for further kinetic and thermodynamic studies.

In Table 1, the adsorption capacity, desorption capacity, and desorption rate of CNPC1 and CNPC2 are all higher than those of other activated carbons. The difference in adsorption capacity may be due to the fact that the carbon source of CNPC1 and CNPC2 is citrus processing wastes. It is shown that the adsorption/desorption capacity of carbon materials is related to their pore structure and functional groups on the surface of materials, and they interact with organic molecules through non-covalent bond forces, such as π-π conjugate action, van der Waals force, hydrogen bond, electrostatic action, and hydrophobic action. CNPC1 and CNPC2 from citrus processing wastes may have more diverse functional groups than other activated carbons, and the affinity between these functional groups and PMF molecules may be stronger than others, so the adsorption/desorption capacity of these two carbons were better than those of others.

However, CNPC1 and CNPC2 are also derived from citrus processing wastes, but their adsorption capacity is different, which may be due to the difference in pore structure caused by different preparation processes. After activation, CNPC1 was freeze-dried, while CNPC2 was dried at 40 °C. In the process of drying, vacuum freeze-drying can keep the physical structure of samples to the greatest extent without destroying the properties of samples. The effect of thermal drying on the sample is the inverse change in temperature and humidity; to a certain extent, this will cause surface contraction. At the same time, the change in temperature will cause the vaporization of internal water and capillary tube expansion, resulting in a change in the sample structure. Therefore, the pore structure of CNPC2 may be changed due to drying in the preparation process, such as pore collapse, thus affecting its adsorption/desorption capacity.

### 3.3. Adsorption Kinetics and Isotherms

**Figure 2:**
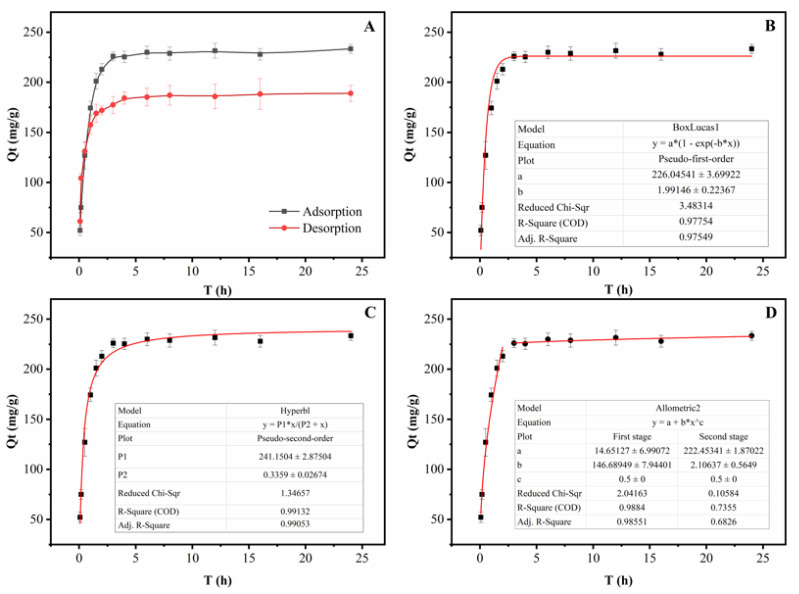
Adsorption/desorption kinetics for the total polymethoxyflavones on CNPC1 (**A**) and the simulation of PMF adsorption into the CNPC1 using the pseudo-first-order kinetic model (**B**), pseudo-second-order kinetic model (**C**), and intraparticle diffusion model (**D**).

The kinetics of adsorption that describe the solute uptake rate governing the contact time of the sorption reaction are one of the important characteristics that define the efficiency of adsorption. Hence, in the present study, the adsorption kinetics of the total PMFs were determined to understand the adsorption behavior of CNPC1. The static kinetic curve obtained is shown in Figure 2A. The adsorption process exhibited three stages. In the first stage, the adsorption capacity showed a linear and rapid increase over the first 1.5 h, but in the second stage the adsorption capacity of CNPC1 increased slowly and reached an adsorption equilibrium at 3 h. This fast adsorption indicated the high affinity between the CNPC1 surface and PMFs. There were also a large number of pore structures on the CNPC1 surface, which provided enough adsorption sites. As the adsorption sites were occupied, the adsorption rate gradually decreased until it reached equilibrium. In addition, in the process of static desorption it was desorbed rapidly and reached equilibrium within 1.5 h. Thus, based on its ability to quickly adsorb/desorb PMFs, CNPC1 was suitable for enriching the total PMFs.

To elucidate the adsorption behavior and mechanism of CNPC1, three kinetics models were adopted to evaluate the adsorption process (Figure 2B–D). The equations and parameters of the three kinetic equations were summarized in Table S2. The adsorption process of the PMFs on CNPC1 was elaborated by the pseudo-second-order kinetics model with a good correlation (R^2^ = 0.9913) in Figure 2C. The results exhibited that the adsorption process of PMFs on CNPC1 was well explained by the pseudo-second-order model. The linear curve of the intra-particle diffusion model does not cross the origin (Figure 2D), which indicates that intra-particle diffusion is not the only speed-controlling step for CNPC1 to adsorb PMFs. Generally, this process can be divided into the two stages, including liquid film diffusion (the adsorbate is transferred from the boundary liquid film to the adsorbent surface) and internal diffusion (the transport of the adsorbate from the adsorbent pores to the adsorption site). Comparing the value of R^2^ of the two stages, it can be seen that the liquid film diffusion equation fits better, so the CNPC1 adsorption of PMFs is mainly limited by liquid film diffusion. Therefore, during the adsorption process PMFs were initially transported to the surface of CNPC1 and then rapidly diffused into the pores of the material.

**Figure 3:**
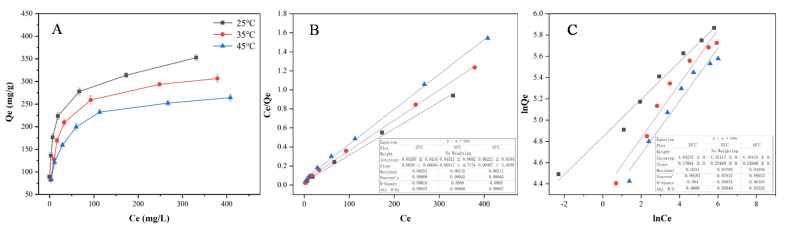
Adsorption isotherms of PMFs on CNPC1 at different temperatures (**A**) and the liner fitting of the Langmuir (**B**) and Freundlich thermodynamic models (**C**).

Additionally, in order to understand the adsorption properties of PMFs on CNPC1, the equilibrium adsorption isotherms on CNPC1 were investigated by varying the initial concentration of PMFs and temperature. The adsorption capacity increased upon decreasing the temperature and increasing the concentration of PMFs (Figure 3A). The results revealed that the adsorption process of PMFs on CNPC1 was intrinsically exothermic. Several models can be used to depict the experimental adsorption isotherms. Herein, the relationship between the adsorption capacity of PMFs on CNPC1 and the PMF equilibrium concentration in the solution was examined using the Langmuir and Freundlich models.

The parameters of the isotherm models are summarized in Table S3 and the liner fitting analysis is shown in Figure 3B,C. The Langmuir equation accurately described the adsorption of PMFs on the CNPC1 with R^2^ values > 0.99, indicating that CNPC1 is fit for the adsorption of PMFs from the extraction solution. The Langmuir isotherm model described monolayer adsorption on a homogeneous surface with no interaction between the adjacent adsorbed molecules. The value of the Langmuir constant K_L_ (K_L_ > 1) indicates that the isotherm is feasible. In the Freundlich equation, n is one of the characteristic constants. When n < 0.5, it is difficult for the adsorption to occur; when n = 1, the adsorption is irreversible; when n > 1, the adsorption is easy. The values of n at different temperatures in this test are all greater than 1, indicating that PMFs are easily adsorbed by CNPC1. In addition, in the range of 25–45 °C, K_L_, K_F_, and Q_m_ all decrease with the increase in temperature, which also indicates that the adsorption process is exothermic adsorption and the cooling is beneficial to the adsorption of PMFs.

### 3.4. Single-Factor Test Results of Static Adsorption and Desorption

**Figure 4:**
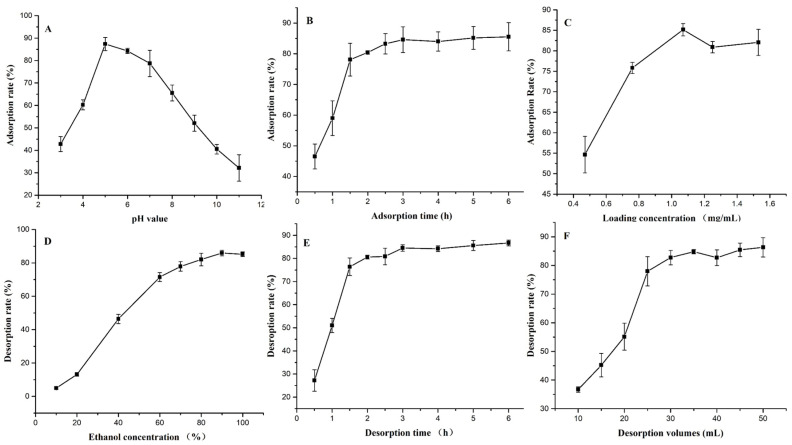
Results of static single-factor tests. (**A**) Effects of the pH value on the adsorption rate; (**B**) effects of the adsorption time on the adsorption rate; (**C**) effects of the loading concentration on the adsorption rate; (**D**) effects of the ethanol concentration on the desorption rate; (**E**) effects of the desorption time on the desorption rate; (**F**) effects of the desorption solution volumes on the desorption rate.

When the other conditions remain unchanged, the effect of the above variables on the adsorption/desorption rate changes, and the results are shown in Figure 4. As can be seen from Figure 4A, the pH of the sample solution has a greater influence on the adsorption rate, and the acidic condition is better than the alkaline condition. The adsorption rate reached the maximum at pH = 5, after which the adsorption rate showed a significant negative correlation with the pH value (linear regression equation was y = −11.102x + 153.25, R^2^ = 0.9898; y = adsorption rate, x = pH value). Therefore, the optimal sample pH range is about 5. The adsorption time had a general effect on the adsorption rate (Figure 4B). The longer the adsorption time within the selected time range, the greater the adsorption amount. However, after reaching 2.5 h the growth trend tends to be flat. Obviously, the optimal adsorption time should be around 2.5 h. In Figure 4C, when the loading concentration was 1.07 mg/mL the adsorption rate was the highest, reaching 85.16%. As a result, 1.07 mg/mL was selected as the best loading concentration. Herein, when the feeding concentration was low, the adsorption ratio increased with the increase in the PMF concentration because the amount of PMFs relative to the active spots became greater and greater. However, with the further increase in the PMF concentration, more impurities were also adsorbed on CNPC1, resulting in competition for active sites between PMFs and impurities; thus, a tendency towards a slight decrease in the adsorption capacity was discovered, which was similar to the enrichment of phenolic acid on X-5 resin reported by Sun L. et al. [33].

It is known that the concentration of desorption fluid has a great influence on the desorption rate. Within the 60% concentration range, the desorption concentration and desorption rate showed an extremely significant positive correlation (correlation coefficient R^2^ = 0.9925), and the linear regression equation was y = 1.3815x − 10.868 (y = desorption rate, x = ethanol concentration). When the concentration of the desorption solution is 90%, the desorption rate is the highest (Figure 4D). Thus, 90% ethanol is the optimal desorption solution. Additionally, when the desorption volume is within the range of 25 mL, the desorption rate increases with the ratio, then tends to be flat, reaching its maximum at 50 mL (86.34%). Therefore, the optimal desorption liquid volume range is 25–50 mL (Figure 4E). It can be seen that the desorption time has a great influence on the desorption rate (Figure 4F). When the adsorption time was less than 1.5 h, the desorption rate increased with the increase in desorption time, then tended to be flat, and at 3 h, the desorption almost reached a dynamic equilibrium. Therefore, the optimal desorption time range is about 3 h.

### 3.5. Effect of Enrichment

**Figure 5:**
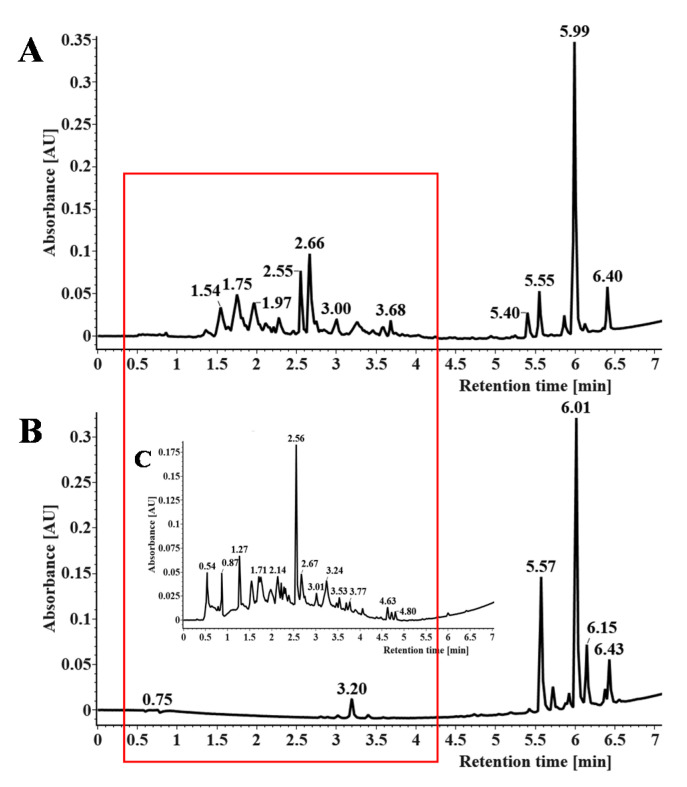
Effects of before- and after-enrichment on the PMF extraction. (**A**) PMF extract solution before and after purification by CNPC1; (**B**) effects of CNPC1 on the ultra-high performance liquid chromatography spectrum of the PMF extract.

The extracts before and after enrichment were characterized by UPLC to further validate the enrichment efficiencies of CNPC1. The UPLC profile of the product before and after enrichment is shown in Figure 5. By comparison, it was found that the purity of PMFs in the resulting extract was 72.01% and the recovery of PMFs by CNPC1 was 74.12%. In Figure 5A, the crude extract of PMFs before purification contained other polar flavonoids (1.5–4.5 min), such as flavanones (the illustration in Figure 5B), which were significantly reduced after enrichment. Thus, CNPC1 could effectively enrich PMFs and remove impurities (in Figure 5B). 

### 3.6. Isolation and Identification of PMFs

Based on the literature and previous studies, the MS-directed prep-HPLC method was employed to isolate and purify the PMF compounds simultaneously. The optimal elution conditions for the isolation of PMFs are shown in Figure S2. Each prep-HPLC peak fraction was automatically collected, and the same fractions were combined and concentrated in vacuo. Finally, six PMF compounds were obtained after crystallization.

**Figure 6:**
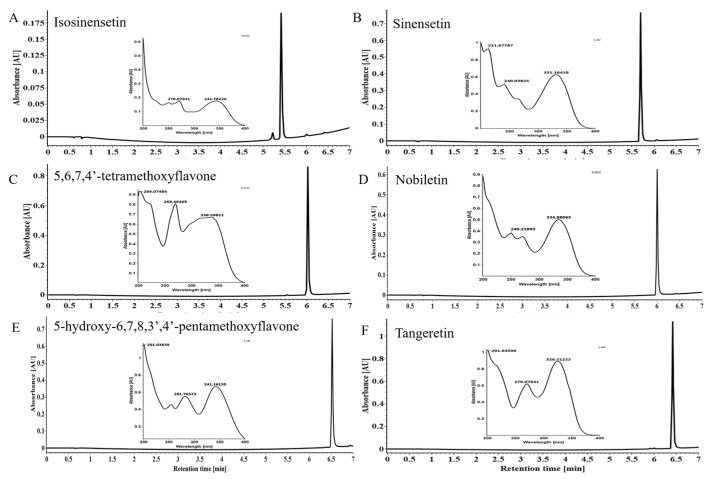
UPLC chromatograms and UV spectrums of isosinensetin (**A**), sinensetin (**B**), 5,6,7,4′-tetramethoxyflavone (**C**), nobiletin (**D**), 5-hydroxy-6,7,8,3′,4′-pentamethoxyflavone (**E**), and tangeretin (**F**).

The obtained compounds (Figure S3 and Figure 6) were identified by UV, MS, and NMR and compared with standards and the literature. The MS and NMR data for these compounds were as follows:

Compound **1** (Figure 6A): brownish yellow powder, ESI–MS: *m*/*z* 373.3287 [M + H]^+^, C_20_H_20_O_7_. ^1^H NMR (400 MHz, CDCl_3_) *δ* 6.84 (s, 1H), 6.68 (s, 1H), 7.53 (s, 1H), 7.14 (d, *J* = 7.9 Hz, 1H), 7.63 (d, *J* = 8.1 Hz, 1H), 3.74 (s, 3H), 3.78 (s, 3H), 3.86 (s, 3H), 3.89 (s, 3H), 3.91 (s, 3H). ^13^C NMR (101 MHz, CDCl_3_) *δ* 175.93 (C–4), 159.57 (C–2), 156.36 (C–7), 155.65 (C–5), 151.07 (C–9), 151.63 (C–4′), 148.97 (C–3′), 129.89 (C–6), 123.25 (C–1′), 119.17 (C–6′), 107.93 (C–10), 111.82 (C–5′), 108.89 (C–2′), 106.53 (C–3), 93.62 (C–8), 60.93, 56.31, 56.28, 55.79, 55.60 (5 × OMe). Compound 1 was identified as 5,7,8,3′,4′-pentamethoxyflavone (isosinensetin) from these spectral data and the published literature; its purity was 95.03%.

Compound **2** (Figure 6B) was a yellow powder: ESI–MS *m*/*z* 373.3297 [M + H]^+^, C_20_H_20_O_7_. ^1^H NMR (400 MHz, CDCl_3_) *δ* 7.55–7.48 (m, 1H), 7.33 (s, 1H), 7.28 (s, 0H), 6.97 (d, *J* = 8.5 Hz, 1H), 6.81 (s, 1H), 6.62 (s, 1H), 4.00 (s, 6H), 3.98 (s, 3H), 3.96 (s, 3H), 3.93 (s, 3H). ^13^C NMR (101 MHz, CDCl_3_) *δ* 177.25(C–4), 161.18(C–2), 157.69(C–7), 154.49(C–5), 152.57(C–9), 151.84(C–4′), 149.28(C–3′), 140.37(C–6), 124.09(C–1′), 119.60(C–6′), 112.83(C–10), 111.16(C–5′), 108.71(C–2′), 107.33(C–3), 96.25(C–8), 62.17, 61.52, 56.31, 56.13, 56.07 (5 × OMe). The above results were consistent with the data for sinensetin; its purity detected by UPLC was 98.33%. 

Compound **3** (Figure 6C) was a yellow powder: ESI–MS *m*/*z* 343. 3384 [M + H]^+^, C_19_H_18_O_6_. ^1^H NMR (400 MHz, CDCl_3_) *δ* 7.93–7.85 (m, 2H), 7.06–6.97 (m, 2H), 6.60 (s, 1H), 6.44 (s, 1H), 4.01 (s, 3H), 3.99 (s, 3H), 3.96 (s, 3H), 3.92 (s, 0H), 3.91 (s, 0H), 3.89 (s, 3H), 3.83 (d, *J* = 1.9 Hz, 0H). ^13^C NMR (101 MHz, CDCl_3_) *δ* 177.94 (C–4), 162.18(C–2), 160.76(C–4′), 156.48(C–7), 156.29(C–5), 151.95(C–9), 130.79(C–6), 127.70(C–2′), 123.83(C–1′), 114.45(C–5′), 108.99(C–10), 106.90(C–3), 92.60(C–8), 61.56, 56.55, 56.29, 55.47 (4 × OMe). The above data were in accordance with the spectral data and physical properties of 5,6,7,4′-tetramethoxyflavone as reported, and its purity was 95.27%.

Compound **4** (Figure 6D) was a white powder: ESI-MS *m*/*z* 403. 1413 [M + H]^+^, C_21_H_22_O_8_. ^1^H NMR (400 MHz, CDCl_3_) *δ* 7.58 (dd, *J* = 8.4, 2.0 Hz, 1H), 7.42 (d, *J* = 2.0 Hz, 1H), 7.00 (d, *J* = 8.5 Hz, 1H), 6.66 (s, 1H), 4.11 (s, 3H), 4.03 (s, 3H), 3.98 (s, 3H), 3.97 (s, 3H), 3.96 (s, 6H). ^13^C NMR (101 MHz, CDCl_3_) *δ* 177.34(C–4), 161.13(C–2), 151.99(C–4′), 151.46(C–7), 149.32(C–3′), 148.41(C–5), 147.71(C–9), 144.12(C–6), 138.00(C–8), 123.99(C–1′), 119.67(C–6′), 114.78(C–10), 111.27(C–5′), 108.63(C–2′), 106.81(C–3), 62.26, 61.96, 61.81, 61.66, 56.09, 55.98 (6 × OMe). These data were in agreement with the values in previous literature, so it was identified as nobiletin; the purity reached up to 99.96%. 

Compound **5** (Figure 6E) was a yellow powder: ESI–MS *m*/*z* 389.3838 [M + H]^+^, C_20_H_20_O_8_. ^1^H NMR (400 MHz, CDCl_3_) *δ* 12.54 (s, 1H), 7.59 (dd, *J* = 8.6, 2.1 Hz, 1H), 7.42 (d, *J* = 2.2 Hz, 1H), 7.00 (d, *J* = 8.5 Hz, 1H), 6.62 (s, 1H), 4.12 (s, 3H), 3.99 (s, 6H), 3.98 (s, 3H), 3.96 (s, 3H), 1.32 (s, 1H). ^13^C NMR (101 MHz, CDCl_3_) *δ* 182.95(C–4), 163.91(C–2), 152.98(C–7), 152.48(C–4′), 149.53(C–3′), 149.39(C–9), 145.75(C–5), 136.58(C–6), 132.95(C–8), 123.70(C–1′), 120.14(C–6′), 111.29(C–5′), 108.80(C–2′), 106.99(C–10), 103.98(C–3), 62.05, 61.71, 61.11, 56.12, 56.00 (5 × OMe). These data were consistent with the literature reported previously, so it was identified as 5-hydroxy-6,7,8,3′,4′-pentamethoxyflavone with a purity of 97.86%.

Compound **6** (Figure 6F) was a light yellow powder: ESI–MS *m*/*z* 373.1297 [M + H]^+^, C_20_H_20_O_7_. ^1^H NMR (400 MHz, CDCl_3_) *δ* 7.93–7.84 (m, 2H), 7.07–6.98 (m, 2H), 6.62 (s, 1H), 4.11 (s, 3H), 4.03 (s, 3H), 3.95 (s, 6H), 3.89 (s, 3H). ^13^C NMR (101 MHz, CDCl_3_) *δ* 177.29 (C–4), 162.30 (C–2), 161.21 (C–4′), 151.38 (C–7), 148.38 (C–5), 147.72 (C–9), 144.08 (C–6), 138.08 (C–8), 127.71(C–2′, 6′), 123.83(C–1′), 114.85(C–10), 114.51(C–3′, 5′), 106.65(C–3), 62.24, 62.01, 61.81, 61.64, 55.49 (5 × OMe). These data agreed well with those for tangeretin reported in the literature; its purity was 99.73%.

### 3.7. Comparison with Other Methods

Finally, the proposed method was compared with other previously reported methods. As listed in Table 2, the prep-HPLC method based on CNPC could be used for the separation and purification of PMFs, had higher adsorption/desorption capacities, and obtained more PMF compounds when compared with previous reports. The prepared adsorbent derived from citrus pomace was cheap and could reduce the resource waste and environmental pollution caused by citrus processing wastes. Therefore, this method had the advantages of a high yield, simple preparation, and rapidity, as well as being low-cost and eco-friendly.

Moreover, CNPCs, as the carbon adsorbent, can not only be used for the enrichment of biologically active compounds, but also can be applied to the field of food safety, such as for pesticide residue determination, especially organophosphorus pesticides. Ren K. et al. [36] used orange pomace as a precursor to synthesize citrus nanoporous carbon through the ZnCl_2_ activation method for the detection of organophosphorus pesticides. Compared with other methods reported in the literature [37,38,39,40] (Table S4), Ren K.’s method has the advantages of a high sensitivity, low cost, simple operation, and rapidity, which makes it significantly better than other methods reported. The NPC material synthesized by Ren K. is very similar to the CNPC in this study, which indicated that biomass-derived nanoporous carbon could be further applied to the field of food safety with great prospects.

## 4. Conclusions

In this study, a more comprehensive use of citrus pomace was explored. Different nanoporous carbons (NPCs) were synthesized and screened for their adsorption/desorption property for PMFs. On this basis, our previous MS-directed prep-HPLC method for PMF separation and purification was improved by coupling it with CNPC enrichment. As presented, a citrus nanoporous carbon derived from citrus-processing wastes with a great enrichment efficiency was synthesized, and using the method of CNPC coupled with MS-Directed prep-HPLC, six PMFs compounds, including 5,6,7,4′-tetramethoxyflavone (95.27%), nobiletin (99.96%), tangeretin (99.73%), sinensetin (98.33%), Isosinensetin (95.03%), and 5-hydroxy-6,7,8,3′,4′-pentamethoxyflavone (97.86%), were simultaneously separated and purified. Therefore, the prepared CNPC has a great potential application for the separation and purification of PMFs in citrus fruits. All these results are of great importance for the future full use of citrus processing wastes, especially for the environmentally friendly utilization of the high-added-value compounds.

## 5. Patents

A Chinese patent (a method for the comprehensive utilization of citrus pomace, 202010480592.7) has been applied resulting from the work reported in this manuscript.

## Figures and Tables

**Table 1:** The adsorption and desorption characteristics of total polymethoxyflavones on different adsorbents.

Adsorbents Type	Adsorption Capacity (mg/g)	Desorption Capacity (mg/g)	Desorption Rate (%)
CNPC1	494.64 ± 5.87	435.62 ± 19.49	88.07 ± 6.36
OPC	479.98 ± 9.60	393.72 ± 4.01	82.03 ± 1.75
ZPC	410.02 ± 3.67	344.76 ± 1.51	84.09 ± 1.73
CNPC2	490.84 ± 3.32	419.42 ± 0.40	85.45 ± 0.83
MCNPC	474.80 ± 2.71	383.06 ± 2.51	80.68 ± 0.03
AC	479.85 ± 1.82	405.85 ± 2.66	84.58 ± 1.78

**Table 2:** Comparison of the proposed methods with other methods for enriching PMFs.

Adsorbents	PMFs Number	Adsorption Capacity (mg/g)	Desorption Capacity (mg/g)	Sample	Ref.
D101	2	8.92	8.23	*Citrus reticulata* Blanco	[41]
NKA	4	10.68	8.75	*Bauhinia championii* Benth	[42]
HPD 450	-	14.55	13.22	*Citrus reticulata* Blanco	[20]
AB-8	5	27.1	26.3	*Citrus reticulata* Blanco	[33]
HPD 100	5	271.52	248.17	*Citrus tangerina* tanaka	[14]
HPD 300	5	301.75	274.01	*Citrus tangerina* tanaka	[19]
CNPC1	6	494.64	435.62	*Citrus sinensis* Osbeck	This work

## Supplementary Materials

The following are available online at https://www.mdpi.com/2079-4991/10/10/1914/s1: Figure S1. SEM image of CNPC1 after adsorption and desorption. Figure S2: The optimal elution conditions for separation and purification of PMFs. Figure S3. Six PMFs compounds obtained in this study. ① Isosinensetin (103.6 mg, 95.03%); ② Sinensetin (32.3 mg, 98.33%); ③ 5,6,7,4’-tetramethoxyflavone (15.3 mg, 95.27%); ④ Nobiletin (158.5 mg, 99.96%); ⑤ 5-desmethylnobiletin (23.7 mg, 97.86%); ⑥ Tangeretin (59.3 mg, 99.73%). Table S1: The flavonoid standards used in this study. Table S2: Adsorption kinetics modeling and parameters for PMFs on CNPC1. Table S3: Model parameters for adsorption of PMFs on CNPC1. Table S4: Comparison of different adsorbents for determining organophosphorus pesticides.
