# Supplementary material for: Nano Porous Carbon Derived from Citrus Pomace for the Separation and Purification of PMFs in Citrus Processing Wastes
Source: Nanomaterials (Basel). 2020 Sep 25;10(10):1914. doi: 10.3390/nano10101914 (PMC7600721; doi:10.3390/nano10101914)
Supplement: Supplementary file 1 [file nanomaterials-10-01914-s001.pdf]

Supplementary Material

Nano porous Carbon Derived from Citrus Pomace for the Separation and Purification of PMFs in Citrus Processing Wastes

Zhenqing Li [1,†], Xin Chen[1,†], Lulu Qiu[1], Yu Wang[1], and Zhiqin Zhou [1,2,3]*

1 College of Horticulture and Landscape Architecture, Southwest University, Chongqing 400716, China; zhenqinglee@126.com (Z.L.); chenxinruyi676893@163.com (X.C.); 18696516978@139.com (L.Q.); wyoo20@163.com (Y.W.).

2 The Southwest Institute of Fruits Nutrition, Banan District, Chongqing 400054, China

3 Key Laboratory of Horticulture Science for Southern Mountainous Regions, Ministry of Education, Chongqing 400715, China

* Correspondence: zhouzhiqin@swu.edu.cn; Tel.: +86-023-6825-1047

† These authors contributed equally to this work.

Supplementary caption:

Figure S1: SEM image of CNPC1 after adsorption and desorption.

Figure S2: The optimal elution conditions for separation and purification of PMFs

Figure S3: Six PMFs compounds obtained in this study. ①Isosinensetin (103.6 mg, 95.03%); ②Sinensetin (32.3 mg, 98.33%); ③ 5,6,7,4'-tetramethoxyflavone (15.3 mg, 95.27%); ④ Nobiletin (158.5 mg, 99.96%); ⑤ 5-desmethylnobiletin (23.7 mg, 97.86%); ⑥Tangeretin (59.3 mg, 99.73%).

Table S1: The flavonoid standards used in this study.

Table S2: Adsorption kinetics modeling and parameters for PMFs on CNPC1.

Table S3: Model parameters for adsorption of PMFs on CNPC1.

Table S4: Comparison of different adsorbents for determining organophosphorus pesticides.

Supplementary Figures

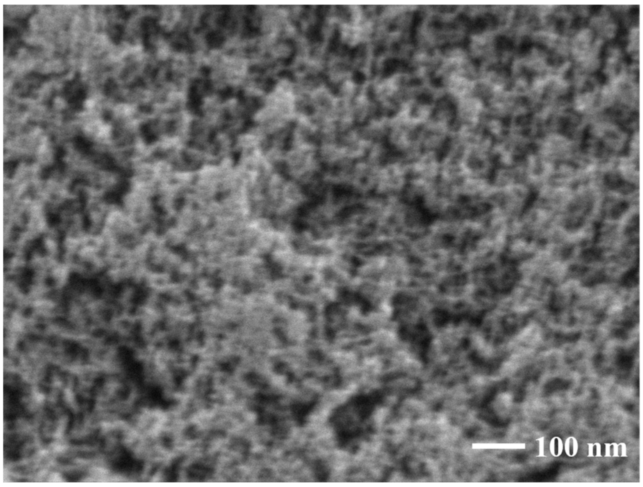

Figure S1. SEM image of CNPC1 after adsorption and desorption.

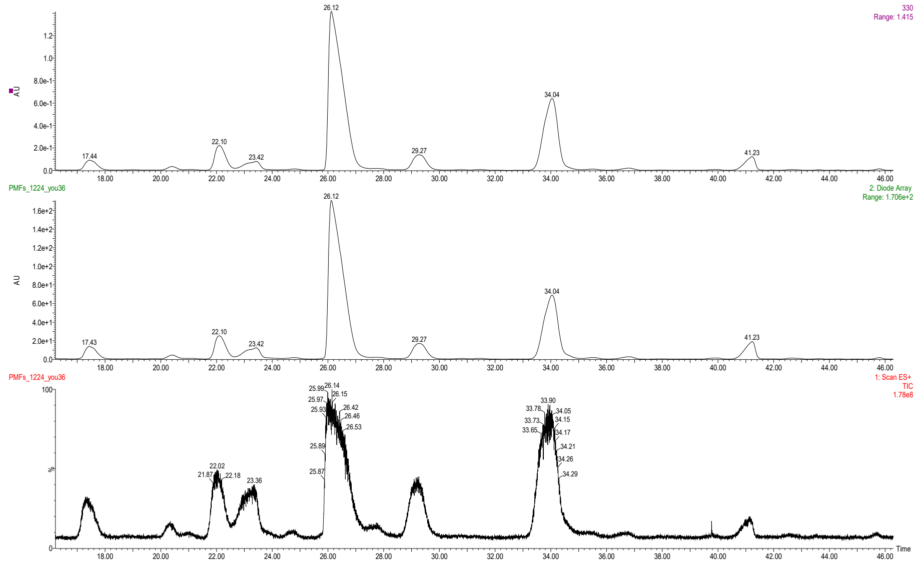

Figure S2. The optimal elution conditions for separation and purification of PMFs.

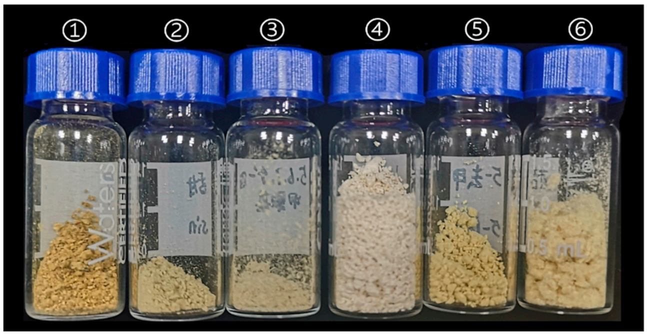

Figure S3. Six PMFs compounds obtained in this study. ①Isosinensetin (103.6 mg, 95.03%); ②Sinensetin (32.3 mg, 98.33%); ③5,6,7,4'-tetramethoxyflavone (15.3 mg, 95.27%); ④Nobiletin (158.5 mg, 99.96%); ⑤5-desmethylnobiletin (23.7 mg, 97.86%); ⑥Tangeretin (59.3 mg, 99.73%).

Supplementary Tables

Table S1. The flavonoid standards used in this study.

No.	Flavonoid	Molecular formula	Molecular mass	CAS No.	Putity	Purchase source
S1	Eriocitrin	$C_{27}H_{32}O_{15}$	596.5321	13463–28–0	≥ 98.0 %	ChromaDex Inc. (Santa Ana, CA, USA)
S2	Rutin	$C_{27}H_{32}O_{14}$	580.5319	14259–46–2	≥ 98.0 %	
S3	Naringin	$C_{21}H_{22}O_{8}$	580.5319	10236–47–2	≥ 98.0 %	
S4	Hesperidin	$C_{28}H_{34}O_{15}$	610.5628	520–26–3	≥ 98.0 %	
S5	Diosmin	$C_{28}H_{32}O_{15}$	608.5433	520–27–4	≥ 98.0 %	
S6	Didymin	$C_{28}H_{34}O_{14}$	594.5674	14259–47–3	≥ 98.0 %	
S7	Neohesperidin	$C_{28}H_{34}O_{15}$	610.5619	13241–33–3	≥ 95.0 %	
S8	Hesperetin	$C_{16}H_{14}O_{6}$	302.2835	520–33–2	≥ 95.0 %	
S9	Naringenin	$C_{15}H_{12}O_{5}$	272.2534	480–41–1	≥ 95.0 %	
S10	Isosinensetin	$C_{20}H_{20}O_{7}$	372.3686	17290–70–9	≥ 98.0 %	
S11	Sinensetin	$C_{20}H_{20}O_{7}$	372.3686	2306–27–6	≥ 98.0 %	
S12	3′,4′,5,7 -Tetrathoxyflavone	$C_{19}H_{18}O_{6}$	342.3403	855–97–0	≥ 97.0 %	
S13	4′,5,6,7 -Tetrathoxyflavone	$C_{19}H_{18}O_{6}$	342.3403	1168–42–9	≥ 98.0 %	ChromaDex Inc. (Santa Ana, CA, USA)
S14	Nobiletin	$C_{21}H_{22}O_{8}$	402.3949	478–01–3	≥ 98.0 %	
S15	3′,4′,3,5,6,7,8-Hetamethoxyflavone	$C_{22}H_{24}O_{9}$	432.4355	1178–24–1	> 98.0%	
S16	4′,5,7-Trimethoxyflavone	$C_{18}H_{16}O_{5}$	312.3277	5631–70–9	≥ 98.7 %	
S17	5-Hydroxy-6,7,8,3′,4′-pentamethoxyflavone	$C_{20}H_{20}O_{8}$	388.3716	2174–59–6	≥ 98.0 %	Chengdu Biopurify Phytochemicals Ltd (Chengdu, China)
S18	Tangeretin	$C_{20}H_{20}O_{7}$	372.3686	481–53–8	≥ 98.0 %	

Table S2. Adsorption kinetics modeling and parameters for PMFs on CNPC1.

Models	Equations	Parameters	Values
Pseudo-first-order model	$Q_t = 226.0454(1 - e^{-1.9915t})$	R^2	0.9775
		Q_e(mg/g)	226.0454
		k_1 (L/min)	−1.9915
Pseudo-second-order model	$Q_t = \frac{715.2882t}{1 + 2.9661t}$	R^2	0.9913
		Q_e (mg/g)	241.1504
		k_2 (g/ (mg·min))	0.0123
Intra-particle diffusion model	$Q_t = 163.6365t^{\frac{1}{2}} + 14.6513$ (first stage)	R^2	0.9884
		k_3 (mg/(g·min$^{1/2}$))	163.6365
		C(mg/g)	14.6513
	$Q_t = 2.1064t^{\frac{1}{2}} + 222.4534$ (second stage)	R^2	0.7355
		k_3(mg/(g·min$^{1/2}$))	2.1064
		C(mg/g)	222.4534

Table S3. Model parameters for adsorption of PMFs on CNPC1.

Temperature	Langmuir			Freundlich		
	Q_{max}	K_L	R^2	K_F	1/n	R^2
25 °C	532.33	5.57	0.9955	126.7479	0.1766	0.9840
35 °C	378.7	2.73	0.9989	75.2789	0.2549	0.9587
45 °C	263.42	0.65	0.9989	65.4574	0.2495	0.9611

Table S4. Comparison of different adsorbents for determining organophosphorus pesticides.

Adsorbents	Samples	Clean-up Time (min)	Determination	LOD (μg/kg)	Ref.
PSA	Fruit juices	> 15	GC-ECD	15–20	[38]
PSA, GCB	Vegetables	9	GC-MS	0.39–8.6	[39]
PSA, GCB, C18	Carrot	9	GC-ECD	0.93–3.38	[37]
MWCNTs	Vegetables, fruits	4	GC-MS	1–20	[40]
NPC	Vegetables, fruits	2	GC-FPD	0.63–5.30	[36]
